# Awareness, Knowledge, and Attitudes Regarding Health Coaching Among Umm Al-Qura University Public Health Students

**DOI:** 10.7759/cureus.48135

**Published:** 2023-11-01

**Authors:** Baraa S Quronfulah, Shatha A Alhasani, Taef S Alzhrani, Rahaf M Babalghith, Lena E Qari, Mohamed O Nour

**Affiliations:** 1 Department of Health Promotion and Health Education, College of Public Health and Health Informatics, Umm Al-Qura University, Makkah, SAU; 2 Department of Public Health and Community Medicine, Damietta Faculty of Medicine, Al-Azhar University, Damietta, EGY

**Keywords:** medical students, health coaching, attitude, knowledge, awareness

## Abstract

Background: In response to the growing burden of chronic diseases on the healthcare system and in pursuit of the health sector goals of Saudi Vision 2030, the Saudi Arabian Ministry of Health implemented an initiative known as health coaching, which helps patients with chronic diseases adopt a healthier lifestyle. This study aimed to assess awareness, knowledge, and attitudes towards the initiative for health coaches among public health students at Umm Al-Qura University in Makkah, Saudi Arabia.

Methods: Data were collected using an online, cross-sectional survey between March 8, 2022 and April 4, 2022. Students from the Health Promotion and Health Education (HPHE) department were compared to students from other public health departments at Umm Al-Qura University. The questionnaire collected data on demographics and awareness, knowledge, and attitudes toward health coaching. Surveys that were at least 80% complete were retained for analysis.

Results: A total of 311 students participated, of which 275 (88.4%) were unaware of health coaching and 156 (50.2%) had insufficient health coaching knowledge. However, 233 (74.9%) had a positive attitude about it. HPHE students scored significantly higher on most items than students from other public health departments.

Conclusion: The students showed positive attitudes but had low scores on awareness and knowledge of health coaching. It is advisable to introduce health coaching into undergraduate medical school curricula. Future research should evaluate students from other health colleges and investigate the effectiveness of health coaching on cost and long-term outcomes of chronic diseases.

## Introduction

Chronic diseases are a major cause of morbidity and mortality worldwide [1]. Lack of awareness may be an underlying factor affecting attitudes and practices regarding the care management of chronic diseases, particularly type-2 diabetes mellitus (T2DM) [2]. Health education about diabetes leads to improvements in knowledge, attitudes, and skills, along with better control of the disease. Such education is widely acknowledged to be an integral component of comprehensive diabetes care [3]. More than one-third of Saudi Arabia’s population has diabetes, which is more prevalent among females than males, at 42% and 37.2%, respectively [4]. The annual cost of treating diabetes in Saudi Arabia is estimated to be 7 billion Saudi riyals (US $1.87 billion) [5].

The government of Saudi Arabia’s ambitious Vision 2030 has three main pillars for achieving its objectives, one of which is to create “a vibrant society” [6]. In order to translate the vision into action, several Vision realization programs were designed, including the Health Sector Transformation Program, which promotes public health by focusing on prevention and improving health awareness through a new model of care (MoC) [7].

Therefore, the Ministry of Health (MOH) is currently undergoing strategic reform in healthcare services delivery, focusing on prevention and primary healthcare (PHC) [8]. The new MoC was adopted by MOH in the health clusters of different regions in Saudi Arabia. This MoC aims to deliver proper care at the right time, in the right place, from the right team to the right patient [9]. Makkah Health Cluster has also launched several initiatives, including health education clinics at several PHC centers [10]. These clinics aim to help patients with chronic diseases cope with their disease and reduce its complications. This service is usually provided by trained health educators who have a bachelor’s degree in public health or health promotion and health education (HPHE). However, some clinics are operated by nurses who received special training in health education for that role.

Health coaching is a practical approach to helping patients manage chronic conditions. It emerged as a possible intervention to help patients adopt health-supportive behaviors that improve quality of life and health outcomes. It involves motivating patients to have the willingness to change their lifestyles and adopt home-based self-care [11]. Health coaching is defined as “a patient-centered process that is based upon behavior change theory and is delivered by health professionals with diverse backgrounds” [12]. The process uses motivational interview counseling to help with positive behavior changes [13]. Motivational interviewing was introduced almost 30 years ago by William Miller and Stephen Rollnick [14]. Since then, hundreds of studies have been published on its effectiveness in health coaching [15-19].

In particular, evidence indicates its effectiveness at reducing hemoglobin A1c (HbA1c) levels [20,21], so the Makkah health cluster’s PHC centers targeted T2DM patients in the first phase of supplying health coaching. Additionally, a recent randomized controlled trial in Makkah revealed promising evidence about the effectiveness of health coaching in reducing HbA1c and improving glycemic control for T2DM patients and suggested diabetes health coaching is vital for facilitating patient self-care, behavior change, and support [2].

Health coaches in PHCs have five principal roles: providing self-management support, bridging the gap between clinician and patient, helping patients navigate the healthcare system (HCS), offering emotional support, and serving as a continuity figure [22]. These roles require training in health coaching to support the delivery of better healthcare. Some medical schools have also introduced health coaching in their medical curricula, training undergraduate students in health coaching for lifestyle and behavior changes [23-27]. Medical students trained in health coaching generally have high levels of acceptance of health coaching for effective behavior change in patients [26,28,29]. A previous Saudi Arabian study reported the positive impacts of these medical students on patient compliance with doctors’ advice [30].

Despite emerging evidence about the effectiveness of health coaching training for medical students, it has yet to be introduced in medical schools’ curricula in Saudi Arabia. The reason is unclear but could be because it can be provided by various healthcare workers, including nurses, educators, and social workers [22]. However, an essential element of public health students’ curricula is learning about behavior change and prevention. Because public health students may be good candidates for health coaching upon graduation, it is essential to understand their current levels of knowledge and attitudes about health coaching.

This study aimed to evaluate the awareness of, knowledge about, and attitudes toward health coaching initiatives at PHC centers in Makkah among a sample of public health students from Umm Al-Qura University (UQU). We expect to benefit public health students by enhancing undergraduate training for health coaching and attracting more students to the career. This will benefit the HCS by delivering public health specialists with health coaching training and by expediting the chronic illness prevention goal of the Vision 2030 Health Sector Transformation Program.

## Materials and methods

Study design and target population

This is a cross-sectional study conducted among undergraduate students from the College of Public Health and Health Informatics (PHHI) at UQU in Makkah.

Study setting

The College of PHHI is a 10-year-old college located at Aziziya campus for male students and at Abdiyah campus for female students in Makkah city. It includes four departments: epidemiology, environmental health, health information management and technology, and health promotion and education. The last two departments include both male and female students.

Sample size calculation

The required sample size was calculated with Cochran’s sample size formula for proportion [31]. The minimum required sample size, calculated with a confidence interval of 95%, margin of error of 5%, and population proportion of 50%, was 385. Because the total student population at the College of PHHI for academic years 2021-2022 was 877, Cochran’s sample size formula modification for a smaller (finite) population was used, giving a sample size of 268. After adding 15% for the non-response rate, a final sample size was calculated to be 308 students.

Data collection method

A self-reported online questionnaire was used to collect primary data.

Questionnaire development and scoring protocol

The research team developed the questionnaire. Firstly, two independent academic researchers experienced with public health had reviewed the questionnaire’s items to ensure they would answer the proposed research question. Then, the modified English version of the questionnaire was translated into Arabic. The questionnaire was then proofread by an independent Arabic language academic before being translated back into English to ensure the wording had not been changed. The questionnaire collected data on the following:

1. Sociodemographic characteristics, such as age, gender, marital status, academic department, and current year of study.

2. Awareness of the health coaching initiative at PHCs in Makkah, measured with five yes/no items.

3. Health coaching knowledge for preventing chronic illnesses measured with eight items answered by yes, no, or “I am not sure”.

4. Attitudes toward health coaching measured by six statements answered on a five-point Likert scale ranging from “strongly disagree” to “strongly agree”. Details of each item were mentioned in the “Results” section.

A standard scoring method was used, giving 1 point for correct and 0 for incorrect answers in the awareness and knowledge sections. In the attitude section, the points given for positive, neutral, and negative options were 2, 1, and 0, respectively. The section scores were 0-5 for awareness, 0-8 for knowledge, and 0-12 for attitude, where higher scores indicating higher levels. The overall awareness, knowledge, and attitudes scores were graded based on modified Bloom's cut-off points; unsatisfactory/negative (≤50%), fair (>50-<80%), and satisfactory/positive (≥80%).

Procedure

Data collection was conducted from March 8, 2022 to April 4, 2022, using the online self-reported questionnaire. A link to the survey was distributed via WhatsApp which is widely used by all students groups. Additionally, flyers including a scannable QR code were distributed on the male and female campuses. An information sheet detailing the study’s purpose and procedure and providing researcher contact information was given in two forms-as a PDF at the survey link or as a hard copy with flyers. Participation consent was assumed with completion of the survey. Participation was voluntary, and it took 3-5 minutes to complete the questionnaire.

Ethical considerations

This study was approved by the Biomedical Research Ethics Committee at UQU (#HAPO-02-K-012-2022-02-971). There was a low risk of harm for participants beyond their time to complete the survey. Maximum efforts were used to protect participants’ anonymity and confidentiality. All data were unidentifiable and stored in a password-protected computer that is only available to the research team.

Statistical analysis

Analysis was conducted with SPSS version 25 (IBM Corp., Armonk, NY). The data were transferred from an Excel spreadsheet to SPSS and then processed, analyzed, and shown in detailed tables. All calculations were performed twice and on different days to eliminate possible calculation errors. Continuous data were reported as means and standard deviations, while categorical data were reported as observation counts and proportions. Fisher’s exact test was used to assess differences in frequencies of qualitative variables, while the independent samples t test was used for the continuous variables. Statistical methods were verified, assuming a significance level of p ≤ 0.05 (two-tailed).

## Results

Demographic characteristics of participants

After contacting all of the students at the College of PHHI, 311 students agreed to participate in the study and submitted a complete questionnaire (35.5% response rate). Participants had a mean age of 20.8 years (SD = 1.2), with 244 (78.5%) females and 67 (21.5%) males. Most were unmarried (97.1%). About two-thirds were in the HPHE department (64%), and almost half (47.3%) were in their second year. The participant demographics are presented in Table 1.

**Table 1:** Demographic characteristics of study participants ^1^Includes epidemiology, environmental health, health information management, and technology departments. HPHE: health promotion and health education.

Characteristic	Total N = 311 (%) mean (± SD)
Age (years)	20.8 (± 1.2)
Gender	
Female	244 (78.5)
Male	67 (21.5)
Marital status	
Single	302 (97.1)
Married	9 (2.9)
Department	
HPHE	199 (64.0)
Other public health departments^1^	112 (36.0)
Academic year	
Second year	147 (47.3)
Third year	83 (26.7)
Fourth year	48 (15.4)
Internship	33 (10.6)

Overall awareness, knowledge about, and attitudes toward health coaching

**Figure 1:**
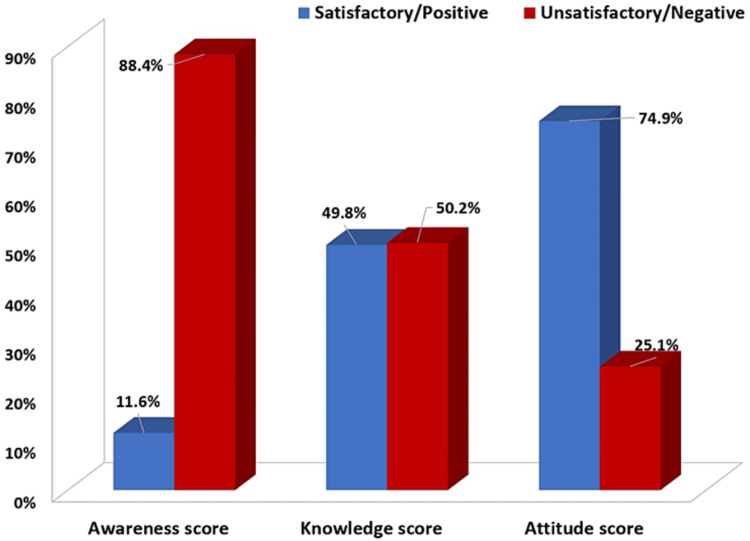
Percentage breakdown of scores for awareness, knowledge, and attitudes on health coaching

Out of 311 participants, a score of at least 80% correct/positive answers was reached by 36 (11.6%), 155 (49.8%), and 233 (74.9%) participants for awareness, knowledge, and attitudes, respectively, on health coaching. This means those participants were considered to have satisfactory awareness, adequate knowledge, and positive attitudes. Conversely, 275 (88.4%), 156 (50.2%), and 78 (25.1%) participants were scored as showing a lack of awareness, unsatisfactory knowledge, and negative attitudes about health coaching, respectively (Figure 1).

Awareness of health coaching

The average total awareness score was 1.37 points (SD = 1.52) out of 5. Comparison of average total awareness scores revealed a statistically significant difference between HPHE department students and students from other public health departments (1.73 ± 1.57 vs. 0.72 ± 1.20; p < 0.001), with HPHE students having significantly higher scores for awareness questions 1, 2, 3, and 5 (Table 2).

**Table 2:** Comparison of groups’ correct answers to awareness items about health coaching Values are presented as numbers and percentages, as analyzed using Fisher exact tests. HPHE: health promotion and health education, PHC: primary healthcare. *Significant.

Variables	HPHE department N = 199 (%)	Other public health departments N = 112 (%)	Total N = 311 (%)	P-value
Aw1: Have you ever heard about health coaching?	122 (61.3)	26 (23.2)	148 (47.6)	<0.001*
Aw2: Do you know there is a health coaching service in Makkah health cluster PHC?	80 (40.2)	17 (15.2)	97 (31.2)	<0.001*
Aw3: Has anyone recommended health coaching benefits to you?	66 (33.2)	14 (12.5)	80 (25.7)	<0.001*
Aw4: Have you or someone you know visited a health coach?	25 (12.6)	10 (8.9)	35 (11.3)	0.357
Aw5: Did you know that you can get health coaching services through Sehhaty application?	51 (25.6)	14 (12.5)	65 (20.9)	0.006 *

Knowledge about health coaching

The average total knowledge score was 6.16 points (SD = 1.63) out of 8. A comparison of average total knowledge scores revealed a significant difference between HPHE department students and students from other public health departments (6.48 ± 1.54 vs. 5.61 ± 1.64; p < 0.001), with HPHE students having significantly higher scores on knowledge items 1, 2, 5, 6, and 8 (Table 3).

**Table 3:** Comparison of groups’ correct answers to health coaching knowledge items Values are presented as numbers and percentages, as analyzed using Fisher exact tests. HPHE: health promotion and health education. *Significant.

Variables	HPHE department N = 199 (%)	Other public health departments N = 112 (%)	Total N = 311 (%)	P-value
Kn1: Is health coaching defined as “a patient-centered process that is based upon behavior change theory and is delivered by health professionals with diverse backgrounds”?	122 (61.3)	38 (33.9)	160 (51.4)	<0.001*
Kn2: Does health coaching help patients with chronic conditions like diabetes control their condition by adopting healthier habits tailored to their lifestyles?	172 (86.4)	81 (72.3)	253 (81.4)	0.004*
Kn3: Is motivational interviewing defined as “a technique used by health coaches to understand their patients’ everyday lives and encourage them to adopt healthier life choices”?	135 (67.8)	67 (59.8)	202 (65.0)	0.174
Kn4: Can health coaches prescribe medication to their patients? REVERSE scoring	186 (93.5)	103 (91.9)	289 (92.9)	0.649
Kn5: Having a health coach has mainly positive effects on people with chronic lifestyle-related diseases, such as cardiovascular disease and diabetes?	185 (93.0)	96 (85.7)	281 (90.4)	0.046*
Kn6: Should a health coach have a good understanding of behavioral change theories and techniques?	185 (93.0)	94 (83.9)	279 (89.7)	0.018*
Kn7: Should health coaches have good communication skills to develop rapport, express empathy, and provide emotional support to their patients?	187 (94.0)	98 (87.5)	285 (91.6)	0.056
Kn8: Should health coaching be delivered by medical or allied health professionals?	117 (58.8)	51 (45.5)	168 (54.0)	0.033*

Attitudes toward health coaching

The average total attitude score was 10.36 points (SD = 1.89) out of 12. A comparison of the average total attitude scores revealed a significant difference between HPHE department students and students from other public health departments (10.87 ± 1.52 vs. 9.46 ± 2.13; p < 0.001), with HPHE students having significantly higher scores for attitude items 1 and 3-6 (Table 4).

**Table 4:** Comparison of groups’ scores for positive attitude items about health coaching Values are presented as numbers and percentages, as analyzed using Fisher exact tests. HPHE: health promotion and health education. *Significant.

Variables	HPHE department N = 199 (%)	Other public health departments N = 112 (%)	Total N = 311 (%)	P-value
At1: Health coaching can improve health-related quality of life and reduce hospital admissions.	188 (94.5)	94 (83.9)	282 (90.7)	0.004*
At2: It is possible to control some health conditions by health coaching only.	121 (60.8)	72 (64.3)	193 (62.1)	0.626
At3: Health coaching services can reduce healthcare costs.	181 (91.0)	81 (72.3)	262 (84.2)	<0.001*
At4: I am interested in learning more about health coaching.	180 (90.5)	90 (80.4)	270 (86.8)	0.014*
At5: I am interested in being a health coach in the future.	159 (79.9)	48 (42.9)	207 (66.6)	<0.001*
At6: I think that I can be a good health coach.	176 (88.4)	69 (61.6)	245 (78.8)	<0.001*

## Discussion

Training undergraduate students in health coaching helps equip them with the necessary knowledge and skills to pursue a career in health coaching after graduation. Attracting more public health graduates to consider a career in health coaching will benefit the HCS by supplying competent health coaches to accelerate the prevention of chronic diseases as the community objective of the new MoC.

Our results revealed that most students at the College of PHHI lacked awareness of and knowledge about health coaching. This may be attributed to the fact that health coaching is a relatively new concept in Saudi PHCs. Consequently, health coaching has not been introduced in medical school curricula in Saudi Arabia. In addition, most participants were in their second year of study, which is the first year of specialization. Thus, they may not have advanced knowledge about disease prevention and behavior change concepts. In contrast, Maini et al. found that third-year medical students trained in health coaching described moving toward a nonjudgmental, solution-oriented mindset. This was reportedly achieved by developing an increased awareness of the importance of health coaching with skill development in person-centered communication, active listening, observation, and self-reflection [29].

This study showed that students have positive attitudes about health coaching, which is similar to a previous study in which medical students reported positive attitudes after engaging in health coaching interventions for older adults with uncontrolled T2DM [28]. However, students in the current study reported positive attitudes even without engaging in health coaching training. This could be explained by their acceptance of health coaching and enthusiasm to learn more about it. Dacey et al. systematically reviewed physical activity counseling in medical school education and found that the programs with the most evidence of improvement were predictive of positive changes in student attitudes about physical activity, counseling knowledge and skills, and self-efficacy in conducting counseling. These programs were likely to have included experiential learning within a theoretical framework [32].

Other undergraduate student curricula progress from traditional shadow learning to opportunities for active engagement in patient care, including health coaching for different lifestyle-related conditions. These can create a new generation of health practitioners who have the necessary awareness and skills to improve and sustain healthy behaviors for themselves and among their patients [23,24,28,33-35].

One interesting finding was that HPHE students scored higher in all three categories than students from other public health departments. This may be due to the study plan and curriculum of the HPHE program including several courses focused on behavioral changes and disease prevention. Those courses introduce behavior change theory and methods of applying health promotion concepts to change beliefs. These focus on the principle of empowerment, which gives strength to individuals and society through decision-making and changing behaviors, and the importance of community participation to bring about changes in public health. Including these concepts in the curriculum could get HPHE students interested in learning more about health coaching.

Study limitations

This study has some limitations that may affect generalization of the results. It is difficult to establish causal inferences with a cross-sectional design. Participation was limited to public health students at UQU and did not include other medical students at the university or from other Saudi universities. Also, most of the participants were second-year female students from the HPHE department. Small sample size and the reliance on self-reported data. The validity and reliability of the questionnaire is a concern. Findings may vary in other populations with different ethnic, cultural, and geographic backgrounds.

Despite its limitations, this study has some strengths. The timing of the study was appropriately in line with the Health Sector Transformation Program in Saudi universities involving updating curricula and study plans. Additionally, this study aligns with the current health coaching program implementation phase at PHC centers in the Makkah health cluster. This study underscores the need to include health coaching concepts and training in medical school curricula, especially for public health students. Having competent trained health coaches will enhance the implementation of the Health Sector Transformation Program of Vision 2030 and aid in achieving its goals [7].

## Conclusions

In conclusion, public health students at UQU had positive attitudes about health coaching, but their awareness of and knowledge about health coaching needs to be improved and further strengthened. Including health coaching in undergraduate medical school, curricula would help students acquire the necessary knowledge and communication skills, including motivational interviewing for changing behaviors; increase student participation in health coaching programs; and allow students to practice and experience the profession in a real-life setting. This will help raise community awareness about the importance of health coaching and sustaining healthy behaviors. Future research on health coaching with larger and more diverse samples should include students from other health colleges and health practitioners. Studies should also investigate the cost-effectiveness of health coaching and its long-term effectiveness with chronic diseases.
